# Implication of gut microbiota metabolites in cardiovascular and metabolic diseases

**DOI:** 10.1007/s00018-018-2901-1

**Published:** 2018-08-12

**Authors:** Francois Brial, Aurélie Le Lay, Marc-Emmanuel Dumas, Dominique Gauguier

**Affiliations:** 1 Sorbonne University, University Paris Descartes, INSERM UMR_S1138, Cordeliers Research Centre, 15 rue de l’Ecole de Médecine, 75006 Paris, France; 2 Section of Biomolecular Medicine, Division of Computational and Systems Medicine, Department of Surgery and Cancer, Faculty of Medicine, Imperial College London, Sir Alexander Fleming Building, London, UK; 3 McGill University and Genome Quebec Innovation Centre, 740 Doctor Penfield Avenue, Montreal, QC H3A 0G1 Canada

**Keywords:** Gut microbiome, Transgenomic interactions, Symbiotic bacterial systems, Metabolic networks, Metabolic modeling, Metabolomics, Microbiota, Complex diseases, Animal models

## Abstract

Evidence from the literature keeps highlighting the impact of mutualistic bacterial communities of the gut microbiota on human health. The gut microbita is a complex ecosystem of symbiotic bacteria which contributes to mammalian host biology by processing, otherwise, indigestible nutrients, supplying essential metabolites, and contributing to modulate its immune system. Advances in sequencing technologies have enabled structural analysis of the human gut microbiota and allowed detection of changes in gut bacterial composition in several common diseases, including cardiometabolic disorders. Biological signals sent by the gut microbiota to the host, including microbial metabolites and pro-inflammatory molecules, mediate microbiome–host genome cross-talk. This rapidly expanding line of research can identify disease-causing and disease-predictive microbial metabolite biomarkers, which can be translated into novel biodiagnostic tests, dietary supplements, and nutritional interventions for personalized therapeutic developments in common diseases. Here, we review results from the most significant studies dealing with the association of products from the gut microbial metabolism with cardiometabolic disorders. We underline the importance of these postbiotic biomarkers in the diagnosis and treatment of human disorders.

## Introduction

The human gut microbiota is a complex system of mutualistic microorganisms, hosting an impressive 100 trillion bacteria from 500 to 1000 species representing 1–3% of body mass and encoding for over 4M genes [1, 2]. It functions as a bioreactor with enormous metabolic capacity and cooperates with the host in many biological functions to form a symbiotic mammalian superorganism [3]. Gut bacteria release bioactive molecules in the gut that can be used by gut mucosal cells or absorbed in the circulation and transported to the liver where they are transformed. The gut microbiota has raised considerable interest due to the possibility of carrying out deep microbiome sequence analysis [4, 5] and to use this information in association studies with various disease conditions. Changes in the architecture of the gut microbiome have been consistently associated with type 2 diabetes and obesity [6–8], which may be accounted for by low microbial gene richness suggesting reduced gut bacterial diversity in patients [9]. Relative resistance to diet-induced obesity and improved glucose tolerance in germ-free rodents also suggest that gut microbiota depletion affects host metabolism and susceptibility to diabetes and obesity [10]. However, the effects of antibiotic-mediated reduction in gut microbiota diversity on host metabolism and insulin sensitivity in humans remain controversial [11, 12].

**Fig. 1:**
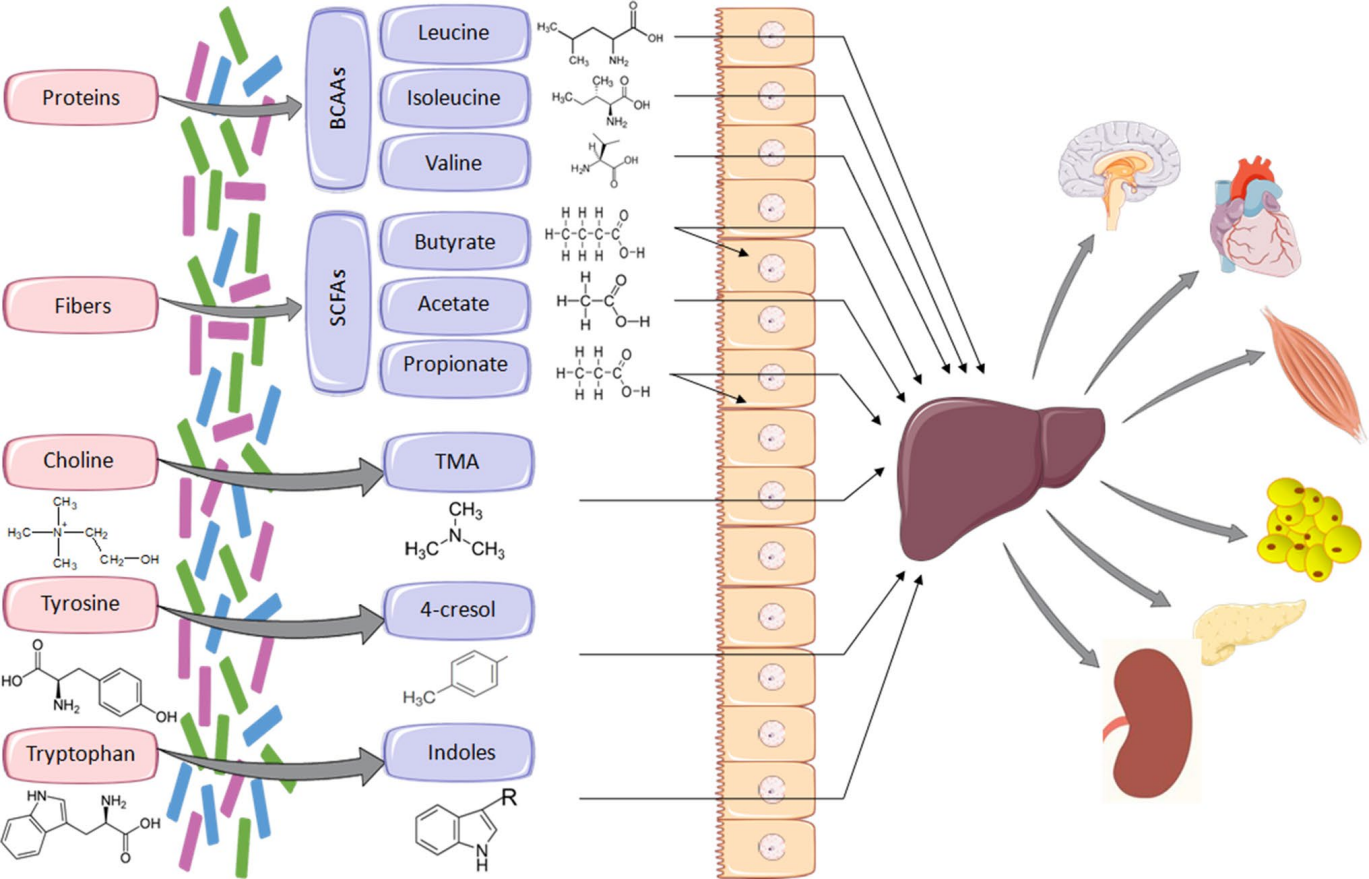
Examples of essential metabolites synthesised by gut bacteria. Metabolites produced by the gut microbiota from dietary substrates are transported to the liver where they can undergo enzymatic modification (e.g., TMA to TMAO), prior to transfer to the circulation and to other organs. SCFAs (predominantly butyrate) can be used locally as an energy source by gut mucosal cells. *BCAAs* branched-chain amino acids, *SCFAs* short-chain fatty acids

The gut microbiota is the central regulator of mammalian fuel intake by processing nutrients into absorbable compounds. It also produces vitamins and essential metabolites that are not synthesised by the host. Changes in the architecture of the gut microbiota are likely to have important repercussion on the regulation of host biochemical pathways and metabolic networks. Many bacterial metabolite end-products of the gut microbiota play crucial roles in the host metabolic homeostasis, immunological processes, and neurobiology, and underline the fundamental importance of this extended genome in human health and disease [13–15]. This article reviews results from association studies of phenotypes relevant to cardiometabolic disorders with products from gut microbial metabolism illustrated in Fig. 1. We do not address intestinal nutrient sensing that triggers humoral and neural responses underlying important gut–brain cross-talk signaling mechanisms in diabetes and obesity, which has been recently reviewed [16] and addressed in landmark papers [17, 18]. This review paper underlines the enormous metabolic capacity of gut bacteria essential for the host and further underscores the importance of high-density metabolic data acquisition from biological samples to address whole-body regulation of biological processes mediated by microbial metabolites.

## Short-chain fatty acids

Short-chain fatty acids (SCFAs) are produced by anaerobic gut bacteria in the caecum and the proximal colon principally through the fermentation of dietary fibers (e.g., nonstarch polysaccharides and low-digestible saccharides), and to a lesser extent of proteins and peptides [19]. The most abundant SCFAs are butyrate, acetate, and propionate [20]. SCFAs are used as an energy source by gut mucosal cells or transferred to the circulation to generate an important source of calory and energy for the organism and to act as signaling molecules. Upon synthesis by the gut microbiota, both propionate and butyrate have local effects as the primary energy source in by gut mucosal cells (butyrate) and by activating intestinal gluconeogenesis (propionate) through distinct mechanisms [18, 21]. Distal effects of SCFAs are illustrated by propionate-mediated stimulation of liver gluconeogenesis, de novo lipid synthesis, and protein synthesis, whereas acetate is a precursor for cholesterol synthesis.

SCFAs have multiple regulatory roles in energy homeostasis, insulin sensitivity, and glucose and lipid metabolism [22]. Association between elevated fecal SCFAs and obesity has emerged from studies in humans [23] and in mice treated with low doses of antibiotics [24]. However, there is a general agreement for their association with reduced risk of cardiovascular and metabolic diseases. Increased plasma insulin in response to propionate was initially demonstrated in healthy volunteers following long-term intracolonic administration of propionate, and was subsequently confirmed in vitro in human islets incubated with propionate [25]. Treatment of overweight individuals with colonic delivery of propionate results in reduced energy intake, adiposity, and lipid liver content, and increased plasma levels of peptide YY (PYY) and glucagon-like peptide 1 (GLP-1) produced locally by enteroendocrine L cells [26]. The effect of propionate on the production of PYY and GLP-1 was confirmed in mouse and rat models following intracolonic propionate administration, and in isolated colonocytes incubated with propionate [27]. Results from an extensive study in obese patients showed that rectal administration of individual SCFAs was associated with increased fasting fat oxidation and energy expenditure, decreased carbohydrate oxidation, and reduced whole-body lipolysis [28]. Further evidence of the beneficial role of microbial SCFAs on human health was obtained in a series of experiments in mice transplanted with gut microbiota from twin pairs discordant for obesity [29]. Mice inoculated with microbiota from lean co-twins showed higher caecal levels of butyrate and propionate than mice inoculated with gut bacteria from obese co-twins, suggesting that capacity to breakdown and ferment polysaccharides into SCFAs is greater in the microbiota from lean individuals than in that of obese patients.

Experiments in animal models have confirmed the associations of SCFAs with host metabolism and diseases. Glucose tolerance and insulin sensitivity are significantly improved in rats treated with diet enriched in butyrate or propionate [18]. Diet supplementation with butyrate in high-fat diet (HFD) fed mice resulted in broad ranging effects, including reduced obesity, glucose intolerance and insulin resistance, enhanced thermogenesis and mitochondrial function, and increased expression of PGC-1α in brown adipose tissue, muscle, and liver, which may be explained by increased activity of AMPK and p38 [30–32]. HFD-induced insulin resistance and obesity in rats is associated with increased plasma concentration of acetate produced by the gut microbiota [17]. Relationship between acetate and phenotypes related to diabetes and obesity is consistent with results from oral administration of acetate in the Otsuka Long Evans Tokushima Fatty (OLETF) rat model of genetically determined obesity and diabetes, which improved glucose tolerance and decreased body weight, hepatic lipid content, and abdominal fat [33].

Data in humans and in experimental models also demonstrated that elevated SCFA can contribute to lowering blood pressure and improving vascular phenotypes. Results from two independent meta-analyses of randomized-controlled trials suggested that increased SCFA induced by probiotics or dietary fiber intake was associated with a reduction in blood pressure in patients with hypertension [34, 35]. More recently, results from the metabolic analyses in the INTERMAP study population showed that 24-h urinary excretion of formate was positively correlated with urinary sodium excretion and inversely associated with both systolic and diastolic blood pressure [36]. Experiments in vivo in mice and in in vitro systems confirmed these observations. Intravenous administration of propionate in vivo in mice lowered blood pressure [37]. Mice fed diet rich in fiber or supplemented with acetate showed a significant reduction in high blood pressure, cardiac fibrosis, and left-ventricular hypertrophy induced by deoxycorticosterone acetate (DOCA) treatment in mice [38]. Repeated intraperitoneal injections of butyrate in mice chronically infused with angiotensin II were able to significantly reduce blood pressure [39]. In vitro experiments showed that butyrate, propionate, and acetate induced dilatation of human colonic resistance vessels [40], and exhibited vasorelaxant properties when tested in isolated rat caudal artery [41].

The cellular mediators of the effects of SCFAs are partly elucidated. Signaling of SCFAs is mediated by the G protein-coupled receptors GPR41 (FFAR3) and GPR43 (FFAR2), predominantly expressed in adipose tissue, intestine, and immune cells [42], as well as GPR109A (HCAR2) [43], and probably other as yet unknown mediators. In a series of elegant experiments in mice and in 3T3-L1 adipocytes, Kimura and colleagues showed that activation of GPR43 by SCFAs results in the inhibition of insulin signaling and reduction of lipid accumulation in fat [44]. On the other hand, another study suggests that relative resistance to obesity in mice-fed HFD supplemented with butyrate and propionate is independent from GPR41 activation [32]. GPR41 is also expressed in the vascular endothelium where it mediates the role of SCFAs in blood pressure regulation [37, 45]. Butyrate also plays an important role in gene expression through its inhibitory effect of histone deacetylases [30], which affects chromatin structure by deacetylation of proteins, and may affect nucleosome positioning [31].

## Methylamines

**Fig. 2:**
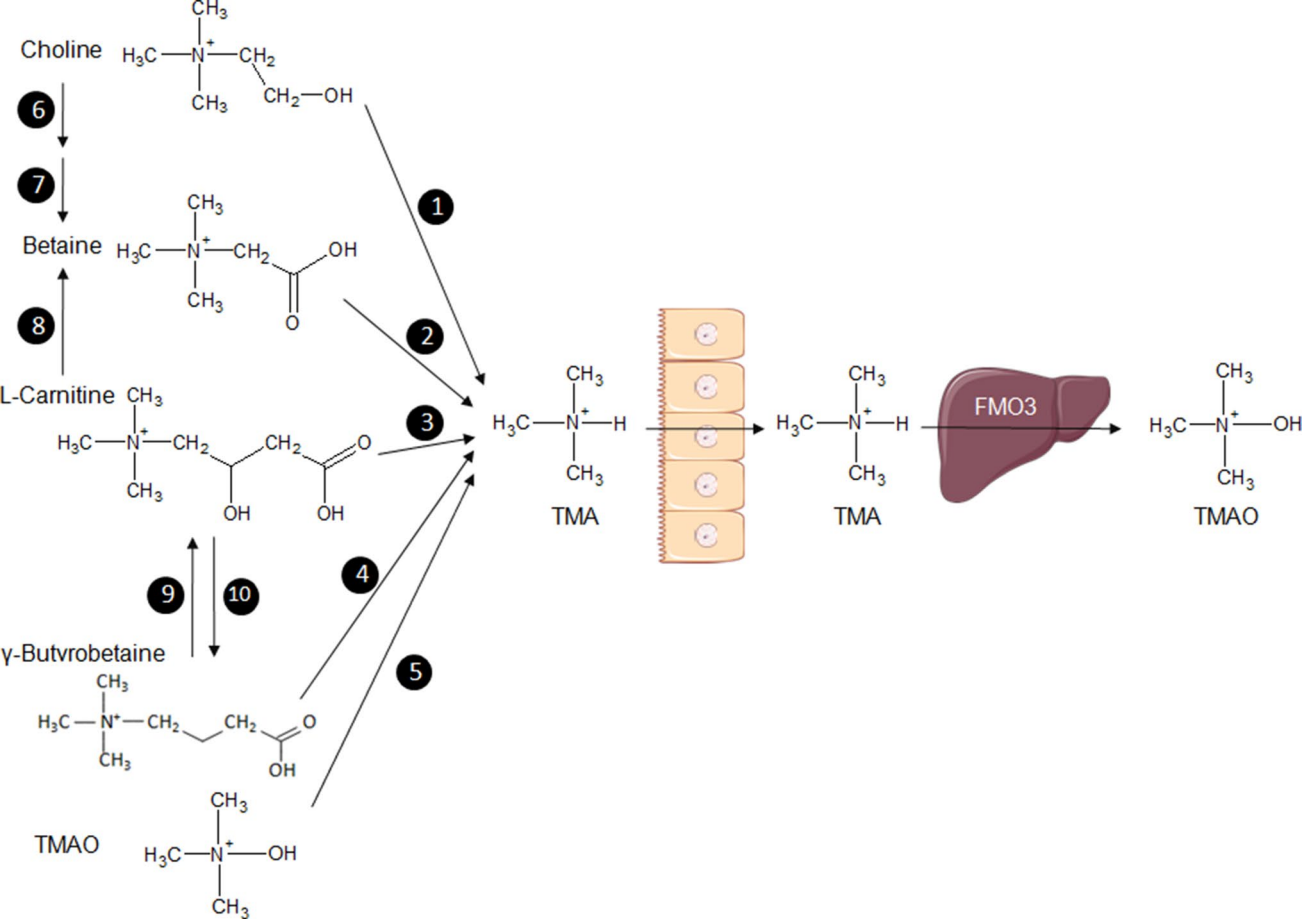
Representation of the methylamine pathway illustrating the microbiota–host co-metabolism. Bacterial enzymes use dietary substrates, choline, betaine, l-carnitine, γ-butyrobetaine, trimethylamine-*N*-oxide (TMAO) to synthesise trimethylamine (TMA), which is transferred across the intestinal endothelium to the circulation and transported to the liver where it is metabolised into TMAO by the enzyme flavin-containing monooxygenase (FMO3). The TMA substrate betaine can be synthesised from choline and l-carnitine. 1: Choline TMA lyase; 2: betaine reductase; 3: carnitine reductase; 4: carnitine TMA lyase; 5: TMAO reductase; 6: choline dehydrogenase; 7: betaine aldehyde dehydrogenase; 8: l-carnitine dehydrogenase; 9: γ-butyrobetaine dioxygenase; 10: γ-butyrobetainyl-CoA: carnitine CoA transferase

Results from extensive studies of the metabolism of choline and methylamines in humans and rodent models are, perhaps, the most compelling illustrations played by the role of gut bacterial metabolites in cardiometabolic diseases. Trimethylamine (TMA) is an amine synthesised from dietary components, l-carnitine, lecithin, choline, and betaine by microbial enzymes (Fig. 2). TMA is oxidised into trimethylamine-*N*-oxide (TMAO) in the liver predominantly by the enzyme flavin-containing monooxygenase 3 (FMO3) [46]. We have recently demonstrated retroconversion of TMA from TMAO by gut bacteria (mostly Enterobacteriaceae) in vivo in mice, which can, therefore, increase the pool of circulating TMA that can be oxidised back to TMAO by FMO3 in the liver [47].

Relationships between TMAO and cardiovascular risk are based on correlative inferences. The multiple detrimental roles of TMAO on human health are still debated [48–50] and thus keep raising interest. For example, elevated plasma levels of TMAO secondary to l-carnitine treatment in ApoE knock-out mice expressing the cholesteryl-ester transfer protein (CEPT) resulted in a reduction of aortic lesions regardless of plasma lipid and lipoprotein levels, and did not alter the formation of macrophage foam cell [51]. The initial observations in humans showing that choline deficiency results in hepatic steatosis reversible by choline supplementation have demonstrated the importance of choline metabolism for the host [52]. Evidence of the pathophysiological impact of choline and methylamines on insulin resistance, fatty liver disease, and obesity was obtained by analyses of plasma and urine metabolomic profiles of mouse strains strongly susceptible (129S6) or resistant (BALB/c) to HFD-induced glucose intolerance, obesity, and fatty liver disease [53]. Fat-fed 129S6 mice exhibited the disruptions of choline metabolism characterised by reduction in plasma levels of phosphatidylcholine and elevated urinary excretion of dimethylamine, TMA, and TMAO. Conversion of choline into methylamines by microbiota in 129S6 mice on HFD resulted in the reduction of the bioavailability of choline.

Subsequent investigations in humans mostly focused on the endpoint of the methylamine pathway (TMAO), which is synthesised by the mammalian metabolism, rather than on its substrate (TMA) synthesised by the gut microbiome. Elevated plasma levels of TMAO have been associated with increased risk of type 2 diabetes mellitus [54], cardiovascular and cerebrovascular diseases [55–57], incident thrombosis risk [58], and carotid intima-media thickness [59] in population-based and intervention studies. Further experiments in mice indicated that elevated levels of circulating TMAO resulted in enhanced aortic atherosclerotic plaque lesions, increased thrombus formation in the carotid artery, perturbed bile acid metabolism, downregulated expression of genes involved in reverse cholesterol transport, and stimulated expression of two macrophage scavenger receptors (CD36, SRA), without significant changes in plasma lipids, glycemia, and hepatic triglycerides [56–58]. In vitro studies showed that TMAO enhanced the function of human platelets and increased platelet adhesion through increased intracellular release of Ca^2+^ and stimulation of inositol-1,4,5-triphosphate signaling [58].

**Fig. 3:**
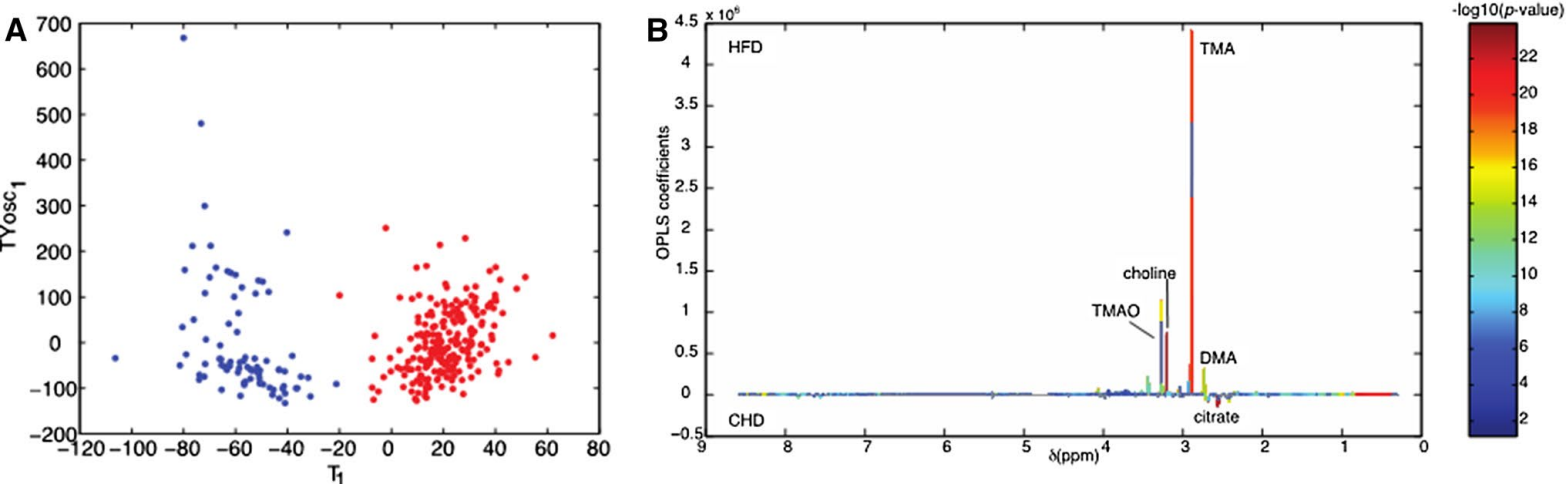
Methylamine-mediated discrimination of adaptation to dietary challenge in isogenic mice. Orthogonal partial least-squares discriminant analysis (O-PLS-DA) scores plots built on 1H NMR metabolomics of 24-h urinary collections was used to discriminate isogenic C57BL/6J mice fed carbohydrate diet (blue) or obesogenic high-fat diet (red) (**a**). Assigned O-PLS-DA model coefficient plot underlines the power of TMA, TMAO and choline to discriminate diet groups (**b**). Adapted from Dumas et al. [60]

Most recently, a study in HFD-fed mice showed that urinary TMAO prior to the dietary challenge is the most significant predictive marker of future heterogeneity in physiological and behavioral anomalies [60]. Variable phenotypic adaptation to HFD in inbred mice is a well-known phenomenon [61, 62], which was recently investigated in groups of HFD-fed isogenic C57BL/6J mice through deep physiological and behavioral phenotyping coupled with the combined analyses of the urine metabolome and adipose tissue transcriptome [60]. Variations in urine levels of choline, TMAO, dimethylamine (DMA), and monomethylamine (MMA) are metabolic markers of adaptation to HFD feeding in isogenic mice (Fig. 3). These changes already exist prior to the dietary challenge and predicted future divergence in disease patterns characterised by various degrees of glucose intolerance and obesity, which correlated with significant divergences in insulin secretion, circulating triglycerides and lipoproteins, and measures of anxiety and activity between extreme responder groups [60]. For example, mice that developed glucose intolerance in response to HFD exhibited a significant increase in urine concentration of products of choline metabolism (e.g., TMA, TMAO, DMA, and dimethylglycine—DMG) prior to HFD-feeding induction, when compared to HFD-fed mice that maintained normal glucose tolerance. This study also showed that chronic subcutaneous infusion of TMAO in HFD-fed mice resulted in paradoxical improvement of both glucose tolerance and glucose-induced insulin secretion, which was confirmed in vitro in isolated pancreatic islets incubated with TMAO. These findings suggest the existence of differences in the composition or the activity of gut bacteria between isogenic individuals prior to any dietary stimulus contribute to the biosynthesis of TMA and predispose to future development of disease phenotypes. The possible implication of the epigenome in this phenomenon is supported by a recent study, showing that depletion of circulating choline in mice caused by intestinal colonisation of choline-utilising bacterial communities results in reduced DNA methylation in several organs and deteriorated metabolic and behavioral phenotypes [63].

Despite growing interest in TMAO biology, its cellular ligands remain unknown. Identification of cellular ligands of TMAO is the next milestone to demonstrate causality with diseases. In contrast, a specific ligand for its precursor TMA has been identified [64]. Following a screening of 42 amines and amine-related compounds, TMA, and to a lesser extent dimethylethylamine (DMEA), activate the trace amine-associated receptor TAAR5, an olfactory G protein-coupled receptor, in a concentration-dependent manner. However, little is known about the biological roles of TMA. Using a mass spectrometry (MS) quantitative assay targeting carnitine, choline, betaine, TMA, and TMAO, a significant association between coronary calcified plaque and cardiovascular risk in humans and serum TMA, and not TMAO, was reported [65]. Urinary levels of TMA, DMA, and DMG, another product of microbial metabolism of choline, have also been associated with Body Mass Index in humans [66]. The existence of multiple TMA substrates (choline, phosphatidylcholine, l-carnitine, betaine, γ-butyrobetaine, and TMAO) and the wide variety of food sources (red meat, fish, egg, and dairy products) that contain TMA or substrates that the gut microbiota can use for TMA biosynthesis, perhaps, prevent systematic nutritional recommendations based on the results obtained until now on methylamines.

## Branched-chain amino acids

The branched-chain amino acids (BCAAs), leucine, isoleucine, and valine, are among the nine essential amino acids synthesised by gut bacteria [67]. They are characterised structurally by aliphatic side-chains with a “branch” (one carbon in the centre linked to three or more carbon atoms). A significant association of the biosynthesis of BCAAs and tryptophan with the bacterial species *Prevotella copri* provided the evidence of functional relationships between changes in the composition of the gut microbiome and these metabolites, as well as potentially indolelactic acid, in insulin-resistant patients [68]. The direct role of BCAAs in the stimulation of insulin secretion [69] and correlations between elevated concentration of plasma BCAAs with obesity and serum insulin [70] have been known for decades. The possible exploitation of BCAAs as biomarkers of cardiometabolic diseases has been addressed in multiple independent patient and population studies, and underlined the complex relationships between circulating BCAAs and disease risk, and the possible implication of confounding factors. Association of plasma BCAAs with insulin resistance was reported in obese [71], non-obese [72], and non-diabetic [68, 73, 74] individuals, and confirmed in twins [75]. Similarly, association between levels of circulating BCAAs and adipokine was evidenced in both diabetic [76] and non-diabetic [74] individuals. Using various study designs and analytical systems, plasma concentrations of all three BCAAs were also found significantly increased in diabetic patients of the German KORA study [77], in female patients for the TwinsUK collection [78], and in a Swedish prospective case control study [79]. Urinary levels of BCAAs were also associated with Body Mass Index in the INTERMAP study [66].

The impact of BCAAs on cardiometabolic phenotypes has been further explored in rodent models and in humans. Addition of BCAAs to HFD in rats reduced the increase in body growth normally caused by HFD, independently from any to significant changes in insulin resistance, which was associated with increased activity of mTOR and JNK [71]. Conversely, dietary restriction in BCAAs improved glucose homeostasis in mice fed control chow diet [80] and reduced adiposity and glucose intolerance induced by HFD in mice [81]. Inoculation of gut microbiota prepared from twin pairs discordant for obesity in mice showed that BCAAs were more elevated when the donor was the obese co-twin, leading to an obese phenotype in transplanted mice, than when the donor was the lean co-twin [29].

Evidence for a causal role of BCAAs in human diabetes was suggested in longitudinal and genetic studies. 12-year follow-up and prospective studies in normoglycemic individuals demonstrated that BCAAs individually and in combination predict future development of type 2 diabetes, suggesting that BCAAs may actually assist the identification of individuals at risk of developing the disease [82]. Most recently, extensive statistical analysis based on Mendelian randomisation in several large populations showed association between variables related to insulin resistance (homeostasis model assessment of insulin resistance—HOMA-IR, fasting insulin) and fasting plasma levels of these BCAAs, but failed to identify significant causal effect of BCAAs on either fasting insulin or HOMA-IR [83]. In contrast, the genetic risk score for insulin resistance traits was significantly associated with increased concentration of plasma BCAAs, indicating a causal impact of insulin resistance on circulating BCAAs. In a large-scale genome-wide association study (GWAS), causal relationships between increased type 2 diabetes risk and high levels of BCAAs determined genetically by genetic polymorphisms at five independent genomic regions were further evidenced through Mendelian randomisation in a meta-analysis [84]. The strongest evidence of association was found with BCAA-raising polymorphisms upstream the gene encoding the protein phosphatase PPM1K on chromosome 4q22.1, which activates the mitochondrial branched-chain alpha-ketoacid dehydrogenase (BCKD) and is, therefore, an attractive candidate to explain this genetic association.

## Bile acids

Bile acids are steroid molecules produced in the liver from cholesterol and, subsequently, processed into secondary bile acids by the gut microbiota. Knowledge of the relationship between gut microbiota and bile acid homeostasis stems from experiments in germ-free mice [85], in animals treated with broad-spectrum antibiotics [86] and in gastrectomised mice [87]. The effects of bile acids on the host metabolism are mediated through regulation of the cholesterol 7-α hydroxylase (CYP7A1) and binding to the nuclear farnesoid X receptor (FXR, NR1H4) and a G protein-coupled receptor (TGR5, GPBAR1). The broad ranging biological functions of bile acids can be explained by the expression of these mediators in many organs involved in cardiovascular and metabolic diseases (liver, adipose tissue, skeletal muscle, pancreatic β cells, and heart), as well as in gut epithelial cells. Bile acid metabolism in health and disease and the central roles of FXR and TGR5 in the concomitant regulation of gut microbiota composition, glucose and lipid metabolism, as well as inflammation and energy expenditure have all been recently reviewed [88–90] and are not addressed in detail here. Bile acid metabolism has been more particularly investigated in response to bariatric surgery (e.g., Roux-en-Y gastric bypass; vertical sleeve gastrectomy). There is now compelling evidence that weight independent metabolic benefits and diabetes remission following bariatric surgery in humans and experimental models is associated with changes in FXR signaling [91], increased plasma levels of bile acids and changes in their composition, as well as altered gut microbiota ecology [91–93]. Both modulation of bile acid metabolism and utilisation of FXR- and TGR5-agonists, therefore, represent attractive though challenging, therapeutic options in type 2 diabetes, non-alcoholic fatty liver disease (NAFLD), and non-alcoholic steatohepatitis (NASH) [94–97], which are, nevertheless, complicated by the expression of FXR and TGR5 in many tissues.

## Polyphenols and indole derivatives

Polyphenols are plant metabolites formed by hydroxylated phenolic rings. Fermentation of dietary polyphenols (mainly hydroxycinnamic acids and flavonoids) by the gut microbiota results in the production of small bioactive compounds consisting of phenolic acids, which can be absorbed by the gut. Products of plant polyphenols have been identified experimentally through systems of in vitro fermentation by human colonic microbiota [98, 99] and incubation with primary hepatocytes [99]. Polyphenols affect the gut microbiota architecture and stimulate bacterial production of SCFAs [100], and can, therefore, have indirect beneficial effects on host metabolism. Absorption and transport of phenolic acids to target tissues are reduced in diabetes and obesity in humans and rodent models [101].

Many intestinal bacteria are able to generate phenolic compounds and indoles from dietary amino acids [102, 103]. 4-cresol (p-cresol, 4-methylphenol) is a phenol metabolised from tyrosine by gut bacteria, mostly by Firmicutes (Clostridiaceae, Eubacteriaceae, Lacnospiraceae, Ruminococcaceae, and Staphylococcaceae) and by bacteroidetes, actinobacteria, and fusobacteria. It is also naturally present at low levels in foods (tomatoes, asparagus, cheeses, butter, bacon, and smoked products) and drinks (coffee, black tea, wine, scotch, whiskey, brandy, and rum). As for the metabolism of choline and methylamines discussed above, clinical and fundamental research focused on 4-cresyl sulfate, which is the product of 4-cresol sulfation in the gut mucosa and liver and is a well-known protein-bound uremic toxin associated with chronic kidney disease [102]. However, the metabolic effects of 4-cresol are largely unexplored. 4-cresol inhibits hepatic drug-metabolising cytochrome P450 and UDP-glucuronosyltransferase enzymes in vitro in human liver microsomes [104]. High concentrations of 4-cresol (100–200 μM) inhibit the differentiation of 3T3-L1 preadipocytes into mature adipocytes, induce apoptosis, and decrease glucose uptake [105], thus indicating that 4-cresol metabolism affects the function of other organs than kidney. Vanillic is another example of a product of biotransformation of polyphenols by the gut microbiota, which has the capacity to reduce hyperinsulinemia, hyperglycemia, hyperlipidemia, and hepatic insulin resistance in high-fat diet-fed rats [106].

The role of indole derivatives in cardiovascular and metabolic diseases is emerging from studies in humans. Indole is a signaling molecule produced exclusively by bacterial tryptophanases from the essential amino acid tryptophan supplied in the diet [107]. Indoxylsulfate is a protein-bound uremic toxin formed through hydroxylation of indole in the liver followed by O-sulfation. Serum concentrations of indoxylsulfate are associated with aortic calcification, arterial stiffness, and increased cardiovascular mortality in patients with chronic kidney disease [108]. Furthermore, indoxylsulfate, as well as indoxyl acetate, activate the aryl hydrocarbon receptor (AHR) pathway in primary human aortic vascular smooth muscle cells and promote thrombosis through upregulation of the expression of tissue factor and inhibition of ubiquitination and degradation of tissue factor [109, 110]. Finally, human population studies showed that serum indole propionate was associated with reduced risk of type 2 diabetes and preserved β-cell function, and negatively correlated with low-grade inflammation. Elevated serum indole propionate was associated with dietary fiber intake, consistent with a functional relationship between diet, gut microbial metabolism, and disease risk [111].

## Other metabolites

Benzoic acid is an aromatic carboxylic acid synthesised in the gut through fermentation by colonic microbiota of dietary aromatic compounds, including aromatic amino acids present in plants [98]. It is used as a food preservative (E211), and is often found in sodas and ready-made meals. It is conjugated in the liver with glycine to produce hippuric acid, which is further conjugated and eliminated in the urine. In a genetic study, we identified that elevated levels of plasma benzoic acid in the Goto–Kakizaki rat model of polygenic type 2 diabetes is controlled by a genetic locus containing a deletion in a uridine diphosphate glucuronosyltransferase [112]. This study was the first demonstration of the genetic control of products of microbial metabolism by host genes that contribute to disease susceptibility. Metabolome-wide association studies in humans identified associations between type 2 diabetes and benzoic acid levels in plasma [113, 114] and urine [113]. On the other hand, results from a randomized, cross-over trial in overweight subjects showed that oral administration of sodium benzoic acid had no significant effects on glucose homeostasis [115].

Biosynthesis and excretion of hippuric acid, the product of benzoic acid degradation, have been reviewed in detail [116]. The implication of this product of microbial–mammalian co-metabolism in human diseases was initially obtained in a metabolome-wide association study of the INTERMAP UK cohort, which demonstrated inverse correlation between urine levels of hippuric acid and hypertension [36] and Body Mass Index [66]. Investigations in small groups of patients and lean controls confirmed association between low levels of urinary hippuric acid and obesity [117] and type 2 diabetes [118]. Interestingly, hippuric acid strongly increased in obese or diabetic individuals who underwent bariatric surgery [117, 119], suggesting that this metabolite may represent a clinical biomarker of disease remission. Urinary levels of hippuric acid were decreased in spontaneously obese rats of the Zucker (*fa*/*fa*) strain mutant for the leptin receptor gene when compared to lean controls heterozygous for the *fa* mutation [118]. This observation was replicated in spontaneously hypertensive rats (SHR) [120] when compared to Wistar Kyoto rats. Variations in urinary levels of hippuric acid prior to HFD feeding in isogenic mice were significantly associated with the future development of obesity, suggesting that changes in the architecture and/or function of the gut microbiota predict disease risk independently of genetic variations [60]. Evidence of direct relationships between hippuric acid and the gut microbiome was recently suggested through the observation of a correlation between urine hippuric acid levels and microbiota ecological diversity determined by 16S rDNA motifs frequency [121].

Fermentation of dietary fibers by the gut microbiota can produce other metabolites than SCFAs that are also synthesised by the host. The organic acid succinate is an intermediate in the synthesis of propionate by gut bacteria and also an abundant product of microbial metabolism which is used as a substrate for intestinal gluconeogenesis [122]. A metabolome-wide association study in the INTERMAP population identified an association between urinary levels of succinate and Body Mass Index [66]. Dietary supplementation with succinate in mice improves glucose tolerance, insulin sensitivity, and energy metabolism, and reduces body growth [122].

## Concluding remarks

The gut microbiome sits at the interface between the host, nutritional, and inflammatory environments, and there is growing evidence of a role of bacterial metabolites as biomarkers of pathophysiological features of cardiometabolic diseases, with applications in disease diagnostics and prognosis in precision medicine. Application of high-density metabolite profiling systems is now essential to broaden the spectrum of microbial metabolites simultaneously detected in biological samples, particularly in biofluids (blood, urine), which can be collected in large populations with minimally invasive methods, as well as in extracts from organ biopsies. Regardless of the biological matrix (plasma, serum, urine, cell preparations, and organ extracts) and the technology used (nuclear magnetic resonance spectroscopy and mass spectrometry), metabolomics generates high-resolution spectra for hundreds to thousands of metabolite signals [123, 124], and has enormous potential in systematic metabolic phenotyping [125], with numerous applications in toxicology [126], prediction of treatment outcomes [127, 128], and molecular epidemiology [36, 66]. It is also a powerful system to document in detail the global functional output of changes in the gut microbiome architecture through qualitative and quantitative analyses of the mammalian-microbial co-metabolome [129], to provide novel insights into disease-predictive and disease-associated biomarkers. It is particularly important, since many foodstuff in westernised societies, including ready-made meals, often contain many synthetic and natural additives (e.g., sweeteners, stabilizers, emulsifiers, thickeners, flavor enhancers, and coloring agents) which modify qualitatively and quantitatively the gut microbiota ecosystem, and can have unpredictable beneficial or adverse biological effects in the host. Artificial sweeteners and emulsifiers (carboxymethylcellulose, polysorbate-80) in the diet alter gut microbiota architecture, and promote glucose intolerance, low-grade inflammation, and obesity [130, 131]. Of note, artificial additives may be metabolised by gut bacteria to unforeseen compounds, which can be detected in biological samples only through systematic untargeted metabolomic profiling methods (i.e., analysis of full-metabolomic spectral data).

Mapping the genetic control of microbial metabolites to the human genome is an important perspective, which will improve our understanding of transgenomic regulations [132] and infer causality to diseases when metabolites and disease traits are controlled by the same genetic loci. Ultimately, the effects of microbial metabolites on health can be altered by naturally occurring polymorphisms in host genes encoding proteins (transporters, receptors, and enzymes) that contribute to their metabolism. Positive correlation of circulating TMAO levels with body weight and adiposity in the hybrid mouse diversity panel provides opportunities to identify genes controlling TMAO metabolism [54]. Deciphering the implication of bacterial metabolites on epigenetic regulations, as demonstrated for TMAO and SCFAs, is also an area of promising research to understand the cross-talk between the gut microbiome and the genome of the host [133, 134].

Therapeutic applications of microbial metabolites require full characterisation of their cellular receptors and the underlying signaling pathways, which are in many cases unknown. Finally, potential therapeutic solutions of this research lie in the identification of bacterial ecosystems able to synthesise metabolites that play a beneficial role in the host metabolism, for their inoculation in patients.

## Abbreviations

AHR: Aryl hydrocarbon receptor
BCAAs: Branched-chain amino acids
CEPT: Cholesteryl-ester transfer protein
CYP7A1: Cholesterol 7-α hydroxylase
DMA: Dimethylamine
DMEA: Dimethylethylamine
DMG: Dimethylglycine
FMO3: Flavin-containing monooxygenase 3
FXR: Farnesoid X receptor
GLP-1: Glucagon-like peptide 1
HFD: High-fat diet
MMA: Monomethylamine
MS: Mass spectrometry
NAFLD: Non-alcoholic fatty liver disease
NASH: Non-alcoholic steatohepatitis
OLETF: Otsuka Long Evans Tokushima Fatty
PYY: Peptide YY
SCFAs: Short-chain fatty acids
TMA: Trimethylamine
TMAO: Trimethylamine-*N*-oxide
